# Supplementary material for: Exploring the limits of exercise capacity in adults with type II diabetes
Source: PLoS One. 2025 Sep 9;20(9):e0331737. doi: 10.1371/journal.pone.0331737 (PMC12419639; doi:10.1371/journal.pone.0331737)
Supplement: S1 Table — (DOCX) [file pone.0331737.s001.docx]

**Supporting information S1.**

| **S1 Table.** Differences between insufficient and good quality NIRS groups | | | | |
| --- | --- | --- | --- | --- |
|  | Total  (N=141) | Low Quality  (N=67) | Good Quality  (N=74) | P value |
| ***Demographics*** |  |  |  |  |
| Age (years) | 61.41 ± 10.38 | 61.07 ± 11.61 | 61.72 ± 9.19 | 0.71 |
| Sex (M/F) | 79/62 | 17/50 | 62/12 | **<0.001** |
| Duration of diabetes (years)* | 6.00 ± 9.00 | 6.00 ± 7.25 | 6.00 ± 9.75 | 0.98 |
| ***Medication intake*** |  |  |  |  |
| *Beta-blocker* | 58 (41%) | 35 (52%) | 23 (31%) | **0.01** |
| *Calcium channel blocker* | 29 (21%) | 13 (19%) | 16 (22%) | 0.75 |
| *Diuretics* | 47 (33%) | 24 (36%) | 23 (31%) | 0.55 |
| Lipid-lowering drug | 90 (64%) | 41 (61%) | 49 (66%) | 0.54 |
| Metformin | 119 (84%) | 53 (79%) | 66 (89%) | 0.10 |
| Insulin | 15 (11%) | 6 (9%) | 9 (12%) | 0.54 |
| SGLT2-inhibitor | 33 (23%) | 16 (24%) | 17 (23%) | 0.90 |
| GLP1-agonist | 46 (33% | 20 (30%) | 26 (35%) | 0.50 |
| Sulfamines | 25 (18%) | 12 (18%) | 13 (18%) | 0.96 |
| DPP4-inhibitor | 8 (6%) | 4 (6%) | 4 (5%) | 0.89 |
| ***Blood pressure*** |  |  |  |  |
| Resting SBP (mmHg) | 129.00 ± 14.81 | 127.61 ± 14.47 | 130.25 ± 15.10 | 0.30 |
| Resting DBP (mmHg) | 81.06 ± 9.36 | 79.96 ± 7.48 | 82.06 ± 10.73 | 0.19 |
| ***Smoking status*** |  |  |  |  |
| Non-smoker | 85 (60%) | 47 (70%) | 38 (52%) | **0.02** |
| Smoker | 42 (30%) | 15 (22%) | 27 (36%) | 0.07 |
| Ex-smoker | 14 (10%) | 5 (8%) | 9 (12%) | 0.35 |
| ***Anthropometrics*** |  |  |  |  |
| Body mass index | 30.49 ± 5.18 | 31.39 ± 5.07 | 29.68 ± 5.17 | 0.05 |
| Body weight (kg) | 89.01 ± 18.35 | 86.76 ± 16.70 | 91.14 ± 19.61 | 0.16 |
| Fat mass (%) | 36.05 ± 8.71 | 41.89 ± 7.48 | 31.02 ± 6.21 | **<0.001** |
| Waist circumference (cm) | 108.84 ± 14.51 | 108.94 ± 14.46 | 108.74 ± 14.66 | 0.94 |
| Adipose tissue thickness (mm) – Vastus lateral* | 3.80 ± 1.90  (N=73) | 5.05 ± 3.70  (N=18) | 3.50 ± 1.80  (N=55) | **<0.001** |
| ***Biohumoral data*** |  |  |  |  |
| HbA1c (%) | 6.62 ± 0.93 | 6.56 ± 0.82 | 6.66 ± 1.03 | 0.53 |
| FPG (mmol/L) | 6.82 ± 1.66 | 6.66 ± 1.45 | 6.98 ± 1.83 | 0.25 |
| Hemoglobin (mmol/L) | 8.86 ± 0.89 | 8.45 ± 0.83 | 9.23 ± 0.77 | **<0.001** |
| Creatinine (μmol/L) | 80.46 ± 27.41 | 78.69 ± 26.53 | 82.23 ± 27.41 | 0.35 |
| eGFR (ml/min/1.73m^2^) | 83.08 ± 20.73 | 80.21 ± 23.16 | 85.68 ± 18.03 | 0.12 |
| Total cholesterol (mmol/L) | 3.77 ± 0.90 | 3.88 ± 0.87 | 3.66 ± 0.91 | 0.14 |
| HDL (mmol/L) | 1.32 ± 0.33 | 1.40 ± 0.37 | 1.24 ± 0.27 | **0.003** |
| LDL (mmol/L) | 1.82 ± 0.80 | 1.85 ± 0.77 | 1.79 ± 0.83 | 0.70 |
| Triglycerides (mmol/L)* | 1.21 ± 0.75 | 1.22 ± 0.68 | 1.18 ± 0.81 | 0.47 |
| HOMA-IR* | 5.07 ± 4.85 | 4.79 ± 3.35 | 5.65 ± 6.00 | 0.05 |
| ***CPET data*** |  |  |  |  |
| V0_2_ @ VAT (ml/kg/min) | 1122.53 ± 392.57 | 943.06 ± 268.63 | 1285.01 ± 417.12 | **<0.001** |
| Peak V0_2_ (ml/min) | 1854.98 ± 673.14 | 1542.54 ± 477.49 | 2137.87 ± 701.53 | **<0.001** |
| Peak V0_2_ (ml/kg/min) | 20.89 ± 6.46 | 17.86 ± 4.61 | 23.64 ± 6.68 | **<0.001** |
| Predicted Peak V0_2_ - %  (Gläser – 2010) | 92.05 ± 19.89 | 86.91 ± 18.86 | 96.70 ± 19.77 | **0.003** |
| Peak workload (watt) | 159.47 ± 63.44 | 129.96 ± 45.37 | 186.19 ± 65.84 | **<0.001** |
| Peak HR (bpm) | 142.52 ± 27.41 | 139.63 ± 28.03 | 145.14 ± 26.76 | 0.24 |
| Peak ventilation (L/min) | 76.08 ± 26.94 | 65.57 ± 24.05 | 85.59 ± 26.00 | **<0.001** |
| Peak RER | 1.16 ± 0.10 | 1.15 ± 0.11 | 1.16 ± 0.08 | 0.81 |
| Peak RPE | 16.51 ± 2.14 | 16.50 ± 2.22 | 16.51 ± 2.08 | 0.97 |
| VE/VCO_2_ slope | 29.86 ± 5.50 | 30.59 ± 6.43 | 29.19 ± 4.45 | 0.13 |
| *SBP: Systolic blood pressure; DBP: Diastolic blood pressure; FPG: Fasting plasma glucose; eGFR: Estimated glomerular filtration rate; HDL: High-density lipoprotein; LDL: Low-density lipoprotein; HR: Heart rate; RER: Respiratory exchange ratio; RPE: Rate of perceived exertion; VE/VCO₂: Ventilatory equivalent for CO₂.*  *Significance level was set at p < 0.05. *Data not normally distributed are presented as median ± IQR; Mann–Whitney U test was used.* | | | | |
