# Supplementary material for: Exploring the limits of exercise capacity in adults with type II diabetes
Source: PLoS One. 2025 Sep 9;20(9):e0331737. doi: 10.1371/journal.pone.0331737 (PMC12419639; doi:10.1371/journal.pone.0331737)
Supplement: S2 Table — (DOCX) [file pone.0331737.s002.docx]

**Supporting information S2.**

| **S2 Table.** Near-Infrared Spectroscopy results adjusted for adipose tissue thickness using ANCOVA | | | | |
| --- | --- | --- | --- | --- |
| **NIRS data** | Total  (N=74) | Lowest fitness  (N = 25) | Highest fitness  (N = 25) | P value |
| *During Exercise* | | | | |
| ΔHHb | 12.67 ± 5.93 | 11.42 ± 7.16 | 14.51 ± 4.02 | 0.52 |
| ΔO_2_Hb | 7.20 ± 3.50 | 7.65 ± 3.69 | 7.35 ± 2.97 | 0.45 |
| ΔtHb | 13.29 ± 5.48 | 10.89 ± 4.70 | 16.66 ± 4.73 | **0.005** |
| ΔHb_diff_ | 15.27 ± 8.73 | 15.43 ± 11.46 | 15.64 ± 4.84 | 0.36 |
| *During Recovery* | | | | |
| ΔHHb | 12.03 ± 6.00 | 10.74 ± 6.96 | 13.77 ± 4.51 | 0.51 |
| ΔO_2_Hb | 17.70 ± 7.01 | 14.06 ± 7.25 | 21.32 ± 4.49 | **0.002** |
| ΔtHb | 8.81 ± 3.32 | 6.61 ± 2.78 | 11.14 ± 2.91 | **<0.001** |
| ΔHb_diff_ | 28.65 ± 12.54 | 24.13 ± 14.02 | 33.82 ± 8.36 | **0.002** |
| *HHb: Deoxygenated hemoglobin; O₂Hb: Oxygenated hemoglobin; tHb: Total hemoglobin; Hbdiff: O₂Hb – HHb.*  *The significance level was set at p < 0.05.* | | | | |
