# Supplementary material for: Exploring the limits of exercise capacity in adults with type II diabetes
Source: PLoS One. 2025 Sep 9;20(9):e0331737. doi: 10.1371/journal.pone.0331737 (PMC12419639; doi:10.1371/journal.pone.0331737)
Supplement: Attachment — Submitted filename: Response to Reviewers.docx [file pone.0331737.s005.docx]

02/08/2025

Dear Editor-in-Chief,

On behalf of my co-authors, I am pleased to resubmit our manuscript entitled *“Exploring the limits of exercise capacity in adults with type II diabetes”* for publication in *“PLOS One”.* We would like to thank the reviewers for their valuable time and efforts in reviewing our research article.

As requested, both a marked and clean version are provided, as well as a point-by-point response to the reviewers’ comments.

# **Editor comments**

1. Please ensure that your manuscript meets PLOS ONE's style requirements, including those for file naming. The PLOS ONE style templates can be found at <https://journals.plos.org/plosone/s/file?id=wjVg/PLOSOne_formatting_sample_main_body.pdf> and <https://journals.plos.org/plosone/s/file?id=ba62/PLOSOne_formatting_sample_title_authors_affiliations.pdf>

***Response:***

*We have revised the manuscript to ensure full compliance with PLOS ONE’s style requirements. In doing so, we closely followed both the style templates and the instructions outlined in the online submission guidelines.*

2. Information for Editor:

Note from Nithya Chari ([plosone@plos.org](mailto:plosone@plos.org)):

This manuscript reports a study of data collected during a clinical trial. The main trial is registered at <https://clinicaltrials.gov>. If a statistical review is needed, email [plosone@plos.org](mailto:plosone@plos.org) and PLOS staff will assign one of our statistical advisors ([http://journals.plos.org/plosone/s/advisory-groups#loc-statistical-advisors](http://track.editorialmanager.com/CL0/http:%2F%2Fjournals.plos.org%2Fplosone%2Fs%2Fadvisory-groups%23loc-statistical-advisors/1/010f0197867baaa0-ed7f2c7f-d462-42a5-9472-fabe88b5c352-000000/X_ad57HXpaVO4cxrjTvgIa-s7fKZG6ftBzKceXq76NM=216)).

***Response:***

*We believe that a formal statistical review is not necessary, as all statistical analyses were conducted in consultation with a qualified biostatistician affiliated with our university*. The biostatistician reviewed the methodology, verified the analyses, and ensured the appropriateness of the statistical approaches used in the study.*

 	*Steffen Fieuws: <http://orcid.org/0000-0002-6875-8366>

3. We suggest you thoroughly copyedit your manuscript for language usage, spelling, and grammar. If you do not know anyone who can help you do this, you may wish to consider employing a professional scientific editing service.

The American Journal Experts (AJE) (<https://www.aje.com/>) is one such service that has extensive experience helping authors meet PLOS guidelines and can provide language editing, translation, manuscript formatting, and figure formatting to ensure your manuscript meets our submission guidelines. Please note that having the manuscript copyedited by AJE or any other editing services does not guarantee selection for peer review or acceptance for publication.

Upon resubmission, please provide the following:

- The name of the colleague or the details of the professional service that edited your manuscript
- A copy of your manuscript showing your changes by either highlighting them or using track changes (uploaded as a *supporting information* file)
- A clean copy of the edited manuscript (uploaded as the new *manuscript* file)

***Response:***

*The manuscript has been thoroughly reviewed for language, spelling, and grammar by both the first and the last author. We have carefully revised the text to ensure clarity and readability. We believe the manuscript now meets the necessary language standards. If any language issues are identified, we would be happy to make further revisions as needed.*

*Upon resubmission, we have included both the revised manuscript and supplementary files in versions with (**supporting information* file*) and without (*manuscript* file) tracked changes. In addition, we have modified the figure legend to align with the journal’s style requirements and uploaded it as a *Supporting Information* file.*

4. Please include your full ethics statement in the ‘Methods’ section of your manuscript file. In your statement, please include the full name of the IRB or ethics committee who approved or waived your study, as well as whether or not you obtained informed written or verbal consent. If consent was waived for your study, please include this information in your statement as well.

***Response:***

*We have now provided full details of the ethics committee in the method section. The statement now specifies the full name of the ethics committee (Ethics Committee Research UZ/KU Leuven) (Page 4, Lines104-106):*

*“*Both study protocols adhered to the Declaration of Helsinki and were approved by the Ethics Committee Research UZ/KU Leuven. Before enrolment, all participants provided written informed consent*”*

5. Thank you for stating the following financial disclosure:
This trial received funding from the Scientific Research Foundation of Flanders (FWO – T004420N and FWO – G095221N)

Please state what role the funders took in the study. If the funders had no role, please state: "The funders had no role in study design, data collection and analysis, decision to publish, or preparation of the manuscript."
If this statement is not correct you must amend it as needed.
Please include this amended Role of Funder statement in your cover letter; we will change the online submission form on your behalf.

***Response:***

*We have updated the financial disclosure and it now reads:*

" This trial received funding from the Scientific Research Foundation of Flanders (FWO – T004420N and FWO – G095221N). The funders had no role in study design, data collection and analysis, decision to publish, or preparation of the manuscript."

Furthermore, in accordance with the journal’s style requirements, we have removed the financial disclosure and competing interest statements from the manuscript itself and provided them only in the appropriate sections of the submission system.

6. Please amend your list of authors on the manuscript to ensure that each author is linked to an affiliation. Authors’ affiliations should reflect the institution where the work was done (if authors moved subsequently, you can also list the new affiliation stating “current affiliation:….” as necessary).

***Response:***

*Author affiliations have been updated to meet the Journal style requirement.	The affiliations now accurately reflect the institutions where the work was conducted.*

7. Please include captions for your Supporting Information files at the end of your manuscript, and update any in-text citations to match accordingly. Please see our Supporting Information guidelines for more information: http://journals.plos.org/plosone/s/supporting-information.

***Response:***

*We have now added a heading with ‘Supporting information’ which includes all supporting tables, in accordance with the journal style requirements*

# **Reviewer comments**

## **Reviewer #1**

*We would like to thank the reviewer for taking the time to carefully read and critically evaluate our manuscript. The constructive comments and suggestions have significantly helped us to improve the quality of the work.*

1. Concern 1: Groups not matched by age.
   The age inclusion criteria for this study is very large (35-80 years old), potentially affecting the aerobic fitness comparison between low fitness and high fitness. Indeed, as reported in the Result section, there was a significant difference in age between low versus high fitness groups. Could the lower outcomes in VO2peak, cardiac output (CO), and NIRS-derived muscle oxygenation in the low fitness group have been significantly influenced by age? To what extent age alone would contribute to these findings? Considering the wide range of ages, the Introduction should give some information on how age affects the pathophysiology of type II Diabetes, and this should connect to the purpose of the study (see specific comments below).
   I believe this is an important limitation of this study that will need to be addressed.

***Response:***

*We thank the reviewer for this valuable comment. It is indeed well established in the literature that age has a negative impact on VO₂peak [1–3]. Therefore, we a priori have defined the low and high fitness group using % of predicted VO_2_peak as calculated using the Gläser equation [4]. This formula takes into account age, sex, height, weight and smoking status to express a patient’s actual VO_2_peak as a percentage of the predicted value, allowing for a more accurate comparison of aerobic fitness between individuals of different ages.*

*Despite this, a significant age difference between both fitness groups remained. We believe that this reflects a clinically meaningful pattern. Specifically, these findings highlight that older individuals with type 2 diabetes are more likely to have lower fitness levels compared to sex and age-matched healthy sedentary individuals than their younger peers with T2DM. This is potentially due to the fact that diabetes-related physiological maladaptations increase over time living with diabetes [5–7]. We now added a couple of sentences to highlight this in our discussion (Page 12-13, Lines 259-266)*

*Additionally, in our regression analysis, the contribution of cardiac output and muscle oxygen extraction was evaluated relative to a baseline model that included all five covariates from the Gläser formula (age, sex, height, weight, and smoking status). As such, we believe that we adjusted for the impact of age in evaluating the role of CO versus muscle oxygen extraction.*

*To maintain a clear focus as also asked by reviewer 2, we how now restricted the introduction on our central research question: aiming to better understand the relative contributions of CO and AVdiff. However, we agree with the reviewer that age is an important factor and potential confounder and therefore we have rewritten the discussion and conclusion section to better highlight this (Page 13, Lines 271-273 and Page 16, Lines 337-339).*

2. Concern 2: NIRS assessment and interpretation.
   Measuring the amplitude changes of NIRS parameters always comes with limitations for between group comparisons. A physiological calibration as described by Barstow (2019), would have helped to limit signal contamination due to adipose tissue thickness for continuous wave NIRS devices, such as the one used in this study.

***Response:***

*We fully acknowledge that signal contamination due to adipose tissue thickness (ATT) is a limitation of continuous-wave NIRS systems* [8–10]*. However, a physiological calibration including an arterial occlusion, as described by Barstow (2019) was not feasible in this study population for several reasons* [9]*. First, in individuals with higher tissue volumes in the lower limb, the pressure required for full arterial occlusion can be experienced as painful or even intolerable* [9]*. Second, since NIRS measurements were taken during a maximal exercise test, an occlusion procedure immediately prior could have interfered with test performance and participant comfort.*

*Despite these limitations, we believe that the influence of ATT on our findings was limited, for the following reasons:*

- ***Strict quality control of NIRS signals*** *We carefully included only participants with high-quality NIRS data. This process involved multiple steps:*
  - *We adhered to the manufacturer’s guidelines on TSI fit factor and optode signal quality, excluding any signals that failed to meet threshold criteria.*
  - *All signals were then manually reviewed by two experienced assessors. Any traces that were evaluated as being non-physiological, were excluded and documented in the Supporting Information. Only if all 3 optodes (providing different penetrations depths) were deemed physiological, the measurement was included in the analysis. As such, we unfortunately had to exclude 67 measurements.*
  - *A moving Gaussian filter was applied to reduce signal noise further.
    As a result, 74 high-quality signals (from an initial 141) were retained for analysis.*
- ***Characteristics of the NIRS device***

*The NIRS measurements were performed using the PortaMon system, which has previously been shown to be less sensitive to variations in ATT and optode pressure compared to other commonly used devices such as MOXY* [11]*. This reduced sensitivity is likely due to the larger source-detector spacing in the PortaMon (30–40 mm versus 12.5–25 mm in MOXY), which enables deeper light penetration and more stable readings* [11]*.*

- ***ATT between groups***

*ATT was measured in a standardized way prior to NIRS application, and no significant differences were observed between the low- and high-fitness groups (3.90* ± 1.80 and 3.25 ± 1.38 respectively, p = 0.57)*. Therefore, we anticipate that the impact of ATT on the NIRS parameters would have been quite similar for both groups.*

- ***Absolute vs relative values***

*Physiological calibration is more recommended if a NIRS system is used to provide absolute concentrations of oxy- and deoxy-hemoglobin (such as frequency and time domain systems)* [9]*. Moreover, it has been shown that physiological calibration makes a difference in the absolute number of the signals but does not affect the overall trend of signals* [11]*. In this study, we used a continuous-wave NIRS system and focused on the relative changes from baseline. Thus, even after correcting for ATT, the overall trend would not have been affected.*

*However, we do understand the reviewer ‘s concern and we do recognize the value of including a correction for ATT to further strengthen our findings. Therefore, we now performed an ANCOVA analysis comparing both fitness groups, while including ATT as a covariate. As ATT could only be reliably measured in 55 participants (74%), primarily due to skin adhesion issues, we also performed an ANCOVA using body fat percentage as a covariate, given its significant correlation with ATT in our study population (r=0.34, p = 0.004). These new statistical analyses were applied to all NIRS-derived variables, except for TSI and the HHb/tHb ratio, where a calibration is not required as both numerator and denominator would be similarly affected* [9]*.*

*Both ANCOVA analyses yielded results consistent with the original analysis and no changes were observed in the statistical significance of our findings. We now refer to these supplementary analyses (S2 and S3) in the manuscript (Page 11, Line 222). Because the ANCOVA results were similar to the original analyses, we have retained the original analyses in the main manuscript. However, should the reviewer prefer, we are open to replacing the original analyses in the main analysis with the new ANCOVA results.*

*Additionally, we also added the number of participants in whom ATT could be measured in our table.*

*Lastly, adjustment for ATT or body fat correction was not performed for the repeated measures ANOVA, as these were intended to evaluate interaction effects across the course of the exercise test, and previous literature suggests that ATT does not affect signal trends* [11]*.*

3. In the Introduction, I would suggest adding more information about the effect of age on type II diabetes and on the physiological responses to exercise. If the authors agree, the purpose of the study should also change accordingly to reflect the population being recruited. Age alone can affect both central and peripheral cardiovascular responses to exercise and the authors, in my opinion, should better link their study to the population being investigated.

***Response:***

*Thank you for this valuable comment. We agree that age can influence both the prevalence and progression of type II diabetes, as well as the physiological responses to exercise, including central and peripheral cardiovascular adaptations Yet, as also mentioned in comment 1, to avoid the impact of age both groups were compared based on their percentage of* predicted VO₂peak, calculated using the Gläser formula, *which accounts for age, sex, height, weight, and smoking status [4]. This approach allowed us to take into account the expected effects of age on exercise capacity. As reviewer 2 asked for a better focus in the introduction, we have opted to rewrite the introduction with a specific focus on our central research question: the relative contributions of CO and AVdiff. However, in response to this question, we have rewritten the discussion and conclusion section to better discuss the role of age. (Page 12-13, Lines 259-266, Page 13, Lines 271-273 and Page 16, Lines 337-339).*

4. Lines 9 – 11: I think this portion about the Fick equation should be placed at the beginning of the following paragraph. In the first paragraph it comes a bit out of nowhere.

***Response:***

*We have revised this section so that the statement on the Fick equation is the first sentence of the second paragraph (Page 3, Line 78-80).*

5. Lines 29 – 33: again, in these lines and in general in the entire Introduction section it seems that the authors will compare two matched groups, which is not the case considering that age was not accounted for. I think this should be changed accordingly.

***Response:***

*As mentioned in Comments 1 and 3, both groups were compared based on their percentage of predicted VO₂peak, as calculated using the Gläser formula [4]. While this approach does not involve explicit matching, the formula does correct for five key variables that can influence VO₂peak, including age.*

*To avoid any potential confusion, we have revised the Introduction to remove language that could imply that participants were directly matched. It now reads:*

“Specifically, we aim to determine whether differences in VO_2peak_ between people with T2DM are primarily driven by differences in cardiac output, peripheral oxygen extraction or a combination of both.”

6. Lines 62- 63: Can the authors add the range of work rates increments used for the incremental test?

***Response:***

*The work rates used in this study started at either 10, 20 or 50 watt, with respective increments of 10, 20 or 25 watts per minute. This has now been added to the method section under ‘Cardiopulmonary exercise test (CPET)’ and now reads (Page 5, Lines 127-128).*

“An individualized RAMP protocol was applied, starting at either 10, 20 or 50 watts, with respective increments of 10, 20 or 25 watts per minute, depending on participants' physical status. This approach aimed to achieve an optimal total test duration between 8 to 12 minutes [12].”

7. Similar to the Introduction, the Discussion section should address the effect of age a little bit deeper.
   Additionally, I think that limitations regarding the NIRS discussed above should also be addressed.

***Response:***

*We thank the reviewer and agree that the role of age should be better addressed in the discussion as well as the acknowledgement of the limitation of the NIRS. As mentioned in previous comments, we compared fitness groups based on the percentage of predicted VO₂peak, calculated using the Gläser formula [4].In addition, our regression analysis assessed the contribution of cardiac output and muscle oxygen extraction relative to a baseline model that included the five covariates from the Gläser formula (age, sex, height, weight, and smoking status). This approach allowed us to account for the influence of age when evaluating the added explanatory value of central and peripheral factors.*

*However, although age was not the primary focus of this study, we recognize its important role in determining outcomes such as VO₂peak, cardiac output, and NIRS-derived muscle oxygenation and we have now addressed this more explicitly in the discussion and conclusion section (Page 12-13, Lines 259-266, Page 13, Lines 271-273 and Page 16, Lines 337-339).*

With regard to the limitations of NIRS, as raised in Comment 2, we have now included ANCOVA analyses using both adipose tissue thickness (ATT) and body fat percentage as covariates. In addition, we expanded the limitations section to explain why physiological calibration procedures (such as arterial occlusion) were not feasible in this clinical population and setting. Although the ANCOVA analyses did not alter the statistical outcomes, we acknowledge that results should still be interpreted with caution (Page 15, Lines 326-328)

8. Lines 200 – 204: the authors stated: “In contrast, those with in the higher fitness group maintained O2Hb levels close to baseline value, suggesting that oxygen delivery and demand were more effectively matched. Consequently, this significant greater increased in muscle perfusion is also reflected in a trend towards greater increases in HHb, a marker of oxygen consumption by the muscles, although this was not significant (29,36).”
   This statement is controversial in my opinion. I don’t think that increased muscle perfusion reflects greater HHb (i.e., muscle oxygen extraction). Theoretically it should be the opposite. According to the Fick equation, greater perfusion should decrease HHb. What I see here is that both greater muscle perfusion and the intracellular capacity to utilize oxygen are optimized in the higher fitness group. Greater muscle perfusion cannot "consequently" reflect greater muscle oxygen extraction (i.e., HHb). Muscle oxygen extraction (HHb) reflects the balance between oxygen delivery and utilization (Grassi and Quaresima, 2016). I suggest the authors to reformulate this section.

***Response:***

*We agree that the original statement was inaccurately phrased. What we intended to express is that, since O₂Hb levels remained stable during exercise (i.e. balance between oxygen delivery and utilization) the observed increase in tHb (i.e. muscle perfusion) was accompanied by a parallel increase in HHb. This indeed suggests that the higher fitness group not only exhibited greater perfusion, but also a more effective utilization of oxygen at the muscular level. We have revised the relevant sentence to better reflect this interpretation.*

*This part now reads (Page 13-14, Lines 285-389):*

*“*In contrast, those in the higher fitness group maintained O_2_Hb levels close to baseline value, suggesting that oxygen delivery and demand were more effectively matched. This greater increase in muscle perfusion was accompanied by a parallel increase in HHb, suggesting not only greater perfusion but also more effective oxygen utilization at the muscular level [13,14].*”*

## **Reviewer #2**

*We would like to thank the reviewer for taking the time to carefully read and critically evaluate our manuscript. The constructive comments and suggestions have significantly helped us to improve the quality of the work.*

1. I commend the authors for conducting a difficult study using challenging NIRS interpretations in a pathological condition such as T2DM during exercise. It is impressive to see the sample size and it is unfortunate, I believe, that no other NIRS device or methodology was used to carry out these potentially intriguing measurements. I am, however, confused about the aims/objectives and rationale of this study and I am a bit disappointed in the fact that existing literature about the application of NIRS in clinical populations with high subcutaneous adipose tissue thickness was not included nor known before the execution of this project (Ferrari et al. 2011, Hamaoka et al, 2011 and Celie et al., 2016). In general, I am/was a bit lost in the aims – scientific story you want to tell in the current study presented. Is it the link between central and local muscle perfusion? Is it an analysis of all parameters of the Fick equation, including muscle O2 extraction and consumption (in the mitochondria)? Is it the impact of B blockers (and diuretics) on central and peripheral haemodynamics? Is it something else? I think the authors should review the storyline and address this issue.

***Response:***

*We thank the reviewer for the comments and appreciation of our work and efforts.*

*We acknowledge that certain parts of the original analysis were not fully aligned with the initial research objective as described in the Introduction. Therefore, we have revised both the analyses and the manuscript to better reflect the original aim: to investigate the relative contributions of central (cardiac output) and peripheral (muscle oxygen extraction) components to VO₂peak in this population.*

*Specifically, we have now included ΔHHb/ΔtHb as a volume-corrected marker of oxygen extraction. We selected this parameter based on previous literature showing that HHb alone is influenced by both oxygen extraction and local blood flow* [15]*. Since greater perfusion facilitates greater extraction, HHb cannot be interpreted independently of blood volume* [16,17]*. Therefore, ΔHHb/ΔtHb provides a more valid estimation of muscle oxygen extraction capacity by accounting for variations in blood volume. Additionally, as mentioned in Bartsow et al., the effect of adipose tissue thickness on this marker is limited, as both the numerator and denominator will be affected to a similar degree* [9]*.*

*Furthermore, we performed a new regression analysis with a focus solely on the additional explanatory value of either cardiac output or ΔHHb/ΔtHb beyond traditional demographic and lifestyle factors (as defined in the Gläser formula [4]). We believe this revision, as suggested by the reviewer, indeed improves the clarity and interpretability of the findings and allows us to better answer the original research question.*

*Finally, we have revised the manuscript to ensure a more consistent and coherent narrative throughout the paper and have included to papers to which the reviewer is referring in the discussion section.*

2. In the study objectives, it was written that: ‘This study aims to explore these mechanisms by examining the relative contributions of CO and peripheral oxygen extraction to VO2peak. Specifically, we aim to determine whether differences in VO2peak between individuals with T2DM with the lowest and highest fitness are primarily driven by differences in CO, peripheral oxygen extraction or a combination of both. ‘ In the results nor discussion section, however, I haven’t really found how you handled, calculated, analysed O2 extraction. It seems to me that the authors predominantly focused on the links between central and local heamodynamics or blood flow (CO and muscle perfusion measured by tHb) as it is clearly written accordingly in the conclusion. Hence, I think this manuscript should be rewritten according to clearly defined objectives that are in agreement with the results – discussion and conclusions. I don’t really think that you presented the Fick equation data with regard to a-v O2 difference.

***Response:***

*In line with Comment 1, we have revised the manuscript to ensure that the analyses and discussion are more clearly aligned with the predefined study objectives. Specifically, we have included the ΔHHb/ΔtHb ratio as a volume-corrected marker of peripheral oxygen extraction. This addition allows us to assess the individual contributions of both the central (cardiac output) and peripheral (oxygen extraction) components to VO₂peak, as originally intended.*

3. An important remark and point I want to make is that I think it is crucial to include an analysis about the correlation/regression between subcutaneous adipose tissue and tHb to investigate how big the impact was of anthropometric data on tHb signal or amplitude presented. Do the authors observe a ‘real’ predictive muscle perfusion effect on VO2peak or could it be explained by stronger or weaker amplitudes due to higher or lower subcutaneous adipose tissue. Despite the fact that this point was included in strengths and limitations, I am interested in what the data says (Vastus Lateralis Adipose tissue and tHb data are available.)

***Response:***

*We fully acknowledge that signal contamination due to adipose tissue thickness (ATT) is a limitation of continuous-wave NIRS systems* [8–10]*. However, a physiological calibration including an arterial occlusion, as described by Barstow (2019) was not feasible in this study population for several reasons* [9]*. First, in individuals with higher tissue volumes in the lower limb, the pressure required for full arterial occlusion can be experienced as painful or even intolerable* [9]*. Second, since NIRS measurements were taken during a maximal exercise test, an occlusion procedure immediately prior could have interfered with test performance and participant comfort.*

*In our sample, a significant correlation between vastus lateralis adipose tissue thickness and tHb was present (r=0.59, p<0.001). However, no significant differences in ATT were observed between the low- and high-fitness groups (3.90* ± 1.80 and 3.25 ± 1.38 respectively, p = 0.57)*. Therefore, the impact of ATT on the NIRS parameters would have been similar for both groups.*

*Moreover, it has been shown that physiological calibration is important when using the absolute number of the signals but does not affect the overall trend of signals* [11]*. In this study, we used a continuous-wave NIRS system and focused on the relative changes from baseline. Thus, even after correcting for ATT, the overall trend would not have been affected.*

*However, we recognize the value of including a correction for ATT to further strengthen our findings. Therefore, we performed an ANCOVA analysis comparing both fitness groups, while including ATT as a covariate. As ATT could only be reliably measured in 55 participants (74%), primarily due to skin adhesion, we also performed an ANCOVA using body fat percentage as a covariate, given its significant correlation with ATT (r=0.34, p = 0.004). These analyses were applied to all NIRS-derived variables, except for TSI and the HHb/tHb ratio, where a calibration is not required as both numerator and denominator would be similarly affected* [9]*.*

*Both ANCOVA analyses yielded results consistent with the original analysis and no changes were observed in the statistical significance of our findings. We now refer to these supplementary analyses in the manuscript (Page 7 Lines 174-177 and Page 11, Line 222) and added the results with adjustment body fat and ATT as supplementary tables S2 and S3 in the revised manuscript. Because the ANCOVA results were similar to the original analyses, we have retained the original analyses in the main manuscript. Should the reviewer prefer, we would be open to replacing the original analyses in the main analysis with the ANCOVA results.*

*Additionally, we added the number of participants in whom ATT could be measured*

*Lastly, we opted not to apply ATT or body fat correction in the repeated measures ANOVA, as these were intended to evaluate interaction effects across the course of the exercise test, and previous literature suggests that ATT does not affect signal trends* [11]*.*

4. Considering the fact that B-blockers could also affect muscle perfusion, I was wondering whether no collinearity was found between both parameters in the regression analysis. Are these factors linked to each other? Did you also investigate homo- and heteroscedasticity of the regression data?

***Response:***

*In our sample no significant correlation was present between B-blockers intake and muscle perfusion, as measured by tHb (r = -0.10, p = 0.416). Given the previous comments, we opted to keep a clear objective and therefore no longer included B-blockers intake as a covariate in the regression analysis.*

*Regarding the assumption of homoscedasticity, we assessed this by visually inspecting the residuals versus predicted values plots for each of the four regression models. In all cases, the residuals showed a random and consistent spread around zero, indicating that the assumption of equal error variances was not violated.*

5. Did the authors investigate the impact of diuretics on VO2 peak, CO and muscle perfusion? Taking into consideration the significant different intake between the higher and lower fitness group, it might be interesting to investigate and include in the regression analysis.

***Response:***

*Diuretics was not significantly correlated with either VO_2_peak (r=-0.21, p=0.07), CO (r=-0.16,p=0.26) or muscle perfusion, as measured by tHb (r-0.19,p=0.11).*

*However, we agree that diuretic use could potentially influence CO. For this reason, we performed an additional regression analysis including both components of peak CO (heart rate and stroke volume) to evaluate their relative contributions to VO₂peak. This analysis showed that while both HR and SV were significantly correlated to VO₂peak (r=0.43, p=0.001 and r=0.29, p=0.035 respectively) , HR emerged as the strongest contributor (highest standardized beta: 0.49 compared to 0.21 for SV). Given that diuretics primarily affect SV, whereas beta-blockers mainly affect HR, this finding suggests that beta-blockers may have a greater impact on VO₂peak than diuretics.*

*As previously mentioned, we chose to focus our analysis on the relative contributions of the central and peripheral components to VO₂peak, and therefore no longer included medication intake as a covariate in the regression models.*

*Nonetheless, in the discussion and conclusion section, we now acknowledge that the use of medication may partially explain the lower cardiac output observed in the lower fitness group. However, investigating the direct impact of specific medications on VO₂peak, cardiac output, or muscle perfusion would require additional analyses and goes beyond the purpose of this study.*

6. Hence, as the authors wrote in the conclusion, it is quite relevant to see that B-blockers (and maybe diuretics too) hamper VO2 peak through impacting muscle perfusion, CO and VO2 peak. The regression model present these factors as independent from each other (B-blocker and tHb were included as independent predictors of VO2 peak, but are they independent…?)

***Response:***

*As previously mentioned, we opted to keep a clear objective and therefore no longer included B-blockers intake as a covariate in the regression analysis. However, in the previous model multicollinearity between the included predictors was assessed using variance inflation factors (VIF) and tolerance statistics. These analyses indicated no evidence of multicollinearity.*

*However, the regression model now includes both cardiac output and the ΔHHb/ΔtHb ratio,*

*to evaluate their independent contributions to VO₂peak.*

7. Is there data available about the degree of insulin resistance in both the higher and lower fitness groups? Was HOMA-IR available?

***Response:***

*Data for both plasma insulin and plasma glucose were available, allowing us to calculate HOMA-IR. We have now included HOMA-IR values in the comparison between the low (4.79 ± 3.35) and good (5.65 ± 6.00) quality NIRS groups (see Supplementary Data, Table 1), as well as between the lower (7.06 ± 6.81) and higher (5.53 ± 5.62) fitness groups (Manuscript, Table 1). No statistically significant differences in HOMA-IR were observed between the groups.*

8. In conclusion, I think the authors need to rethink the presentation or the angle of their data/study and align it with clearly defined aims, results, discussion and conclusion. Notwithstanding my critical view towards the current form of the study, I believe that it does, however contain relevant information to publish.

***Response:***

*We have substantially revised the manuscript to ensure a more coherent and focused narrative. Specifically, we have refined the study objective to emphasize the investigation of the relative contributions of central (cardiac output) and peripheral (oxygen extraction)*

*components to VO₂peak in adults with T2DM. We have aligned the statistical analyses accordingly by including both cardiac output and the ΔHHb/ΔtHb ratio as key predictors in our regression models.*

*In addition, we have restructured the results and discussion sections to clearly reflect this focus and have adjusted the conclusion to remain consistent with the refined objective and findings. We trust that these revisions have addressed the concern and improved the clarity and consistency of the manuscript.*

Minor remarks:

1. Line 2-5: Adjust sentence, not correct

***Response:***

*We have revised the sentence, which now reads (Page 3, Line 72-74):*

*“This reduced VO_2_peak is a key factor contributing to adverse clinical outcomes and reduced life expectancy in this population* [18,19]*. An improvement in VO_2_peak by one metabolic equivalent of a task (MET) is associated with a 14-19% reduction in mortality risk* [20,21]

2. Line 177-179: Eliminate Fick equation as muscle perfusion rather than O2 extraction is reported.

***Response:***

Since we have revised the analyses and now include a marker of oxygen extraction (ΔHHb/ΔtHb), we believe it is appropriate to retain this sentence referring to the Fick equation.

3. Line 223-226 : Here the differences in subcutaneous adipose tissue could be a crucial mediator as well… Did you measure real muscle oxy and deoxygenation or lipied oxygenation dynamics?

***Response:***

*We conducted ANCOVA analyses using both adipose tissue thickness (ATT) and body fat percentage as covariates to control for potential confounding effects. Both models yielded results consistent with the original analysis, and no changes in statistical significance were observed. The reasons why ATT may not have had a significant impact on the outcomes are also discussed in our earlier response.*

*Nonetheless, we have now expanded the limitations section to emphasize that, despite the similar ANCOVA results, caution is still warranted when interpreting NIRS data in this population. While ATT between the lowest and highest fitness tertiles were not significantly different, suggesting a minimal impact on group-level comparisons, we acknowledge that a substantial number of participants had NIRS data of insufficient quality. Importantly, these individuals also demonstrated significantly lower VO₂peak values, which limits the generalizability of our findings to the broader T2DM population.*

# **References**

1.	Marcin T, Eser P, Prescott E, Mikkelsen N, Prins LF, Kolkman EK, et al. Predictors of pre-rehabilitation exercise capacity in elderly European cardiac patients – The EU-CaRE study. European Journal of Preventive Cardiology. 2020;27: 1702–1712. doi:10.1177/2047487319894676

2.	Price MJ, Smith PM, Bottoms LM, Hill MW. The effect of age and sex on peak oxygen uptake during upper and lower body exercise: A systematic review. Experimental Gerontology. 2024;190: 112427. doi:10.1016/j.exger.2024.112427

3.	Letnes JM, Nes BM, Wisløff U. Age-related decline in peak oxygen uptake: Cross-sectional vs. longitudinal findings. A review. Int J Cardiol Cardiovasc Risk Prev. 2023;16: 200171. doi:10.1016/j.ijcrp.2023.200171

4.	Sven G, Koch B, Ittermann T, Christoph S, Marcus D, Felix SB, et al. Influence of age, sex, body size, smoking, and β blockade on key gas exchange exercise parameters in an adult population. European Journal of Cardiovascular Prevention & Rehabilitation. 2010;17: 469–476. doi:10.1097/HJR.0b013e328336a124

5.	Nesti L, Pugliese NR, Sciuto P, Natali A. Type 2 diabetes and reduced exercise tolerance: a review of the literature through an integrated physiology approach. Cardiovascular Diabetology. 2020;19: 134. doi:10.1186/s12933-020-01109-1

6.	Petersen KF, Dufour S, Befroy D, Garcia R, Shulman GI. Impaired Mitochondrial Activity in the Insulin-Resistant Offspring of Patients with Type 2 Diabetes. New England Journal of Medicine. 2004;350: 664–671. doi:10.1056/NEJMoa031314

7.	Kim J-A, Wei Y, Sowers JR. Role of mitochondrial dysfunction in insulin resistance. Circulation research. 2008;102 4: 401–14. doi:10.1161/CIRCRESAHA.107.165472

8.	Jones S, Chiesa ST, Chaturvedi N, Hughes AD. Recent developments in near-infrared spectroscopy (NIRS) for the assessment of local skeletal muscle microvascular function and capacity to utilise oxygen. Artery Res. 2016;16: 25–33. doi:10.1016/j.artres.2016.09.001

9.	Barstow TJ. Understanding near infrared spectroscopy and its application to skeletal muscle research. Journal of Applied Physiology. 2019;126: 1360–1376. doi:10.1152/japplphysiol.00166.2018

10.	Niemeijer VM, Jansen JP, Dijk T van, Spee RF, Meijer EJ, Kemps HMC, et al. The influence of adipose tissue on spatially resolved near-infrared spectroscopy derived skeletal muscle oxygenation: the extent of the problem. Physiol Meas. 2017;38: 539. doi:10.1088/1361-6579/aa5dd5

11.	McManus CJ, Collison J, Cooper CE. Performance comparison of the MOXY and PortaMon near-infrared spectroscopy muscle oximeters at rest and during exercise. J Biomed Opt. 2018;23: 1–14. doi:10.1117/1.JBO.23.1.015007

12.	Buchfuhrer MJ, Hansen JE, Robinson TE, Sue DY, Wasserman K, Whipp BJ. Optimizing the exercise protocol for cardiopulmonary assessment. Journal of Applied Physiology. 1983;55: 1558–1564. doi:10.1152/jappl.1983.55.5.1558

13.	Grassi B, Pogliaghi S, Rampichini S, Quaresima V, Ferrari M, Marconi C, et al. Muscle oxygenation and pulmonary gas exchange kinetics during cycling exercise on-transitions in humans. Journal of Applied Physiology. 2003;95: 149–158. doi:10.1152/japplphysiol.00695.2002

14.	DeLorey DS, Kowalchuk JM, Paterson DH. Relationship between pulmonary O_2_ uptake kinetics and muscle deoxygenation during moderate-intensity exercise. Journal of Applied Physiology. 2003;95: 113–120. doi:10.1152/japplphysiol.00956.2002

15.	Grassi B, Quaresima V. Near-infrared spectroscopy and skeletal muscle oxidative function in vivo in health and disease: a review from an exercise physiology perspective. JBO. 2016;21: 091313. doi:10.1117/1.JBO.21.9.091313

16.	Ryan TE, Erickson ML, Brizendine JT, Young H-J, McCully KK. Noninvasive evaluation of skeletal muscle mitochondrial capacity with near-infrared spectroscopy: correcting for blood volume changes. Journal of Applied Physiology. 2012;113: 175–183. doi:10.1152/japplphysiol.00319.2012

17.	Kime R, Fujioka M, Osawa T, Takagi S, Niwayama M, Kaneko Y, et al. Which Is the Best Indicator of Muscle Oxygen Extraction During Exercise Using NIRS?: Evidence that HHb Is Not the Candidate. In: Van Huffel S, Naulaers G, Caicedo A, Bruley DF, Harrison DK, editors. Oxygen Transport to Tissue XXXV. New York, NY: Springer; 2013. pp. 163–169. doi:10.1007/978-1-4614-7411-1_23

18.	Church TS, LaMonte MJ, Barlow CE, Blair SN. Cardiorespiratory fitness and body mass index as predictors of cardiovascular disease mortality among men with diabetes. Arch Intern Med. 2005;165: 2114–2120. doi:10.1001/archinte.165.18.2114

19.	Pandey A, Patel KV, Bahnson JL, Gaussoin SA, Martin CK, Balasubramanyam A, et al. Association of Intensive Lifestyle Intervention, Fitness, and Body Mass Index With Risk of Heart Failure in Overweight or Obese Adults With Type 2 Diabetes Mellitus: An Analysis From the Look AHEAD Trial. Circulation. 2020;141: 1295–1306. doi:10.1161/CIRCULATIONAHA.119.044865

20.	Kokkinos P, Myers J, Nylen E, Panagiotakos DB, Manolis A, Pittaras A, et al. Exercise Capacity and All-Cause Mortality in African American and Caucasian Men With Type 2 Diabetes. Diabetes Care. 2009;32: 623–628. doi:10.2337/dc08-1876

21.	Mandsager K, Harb S, Cremer P, Phelan D, Nissen SE, Jaber W. Association of Cardiorespiratory Fitness With Long-term Mortality Among Adults Undergoing Exercise Treadmill Testing. JAMA Netw Open. 2018;1: e183605. doi:10.1001/jamanetworkopen.2018.3605
